# Adamantinoma: A SEER-based Epidemiological Analysis

**DOI:** 10.1007/s13193-024-02002-z

**Published:** 2024-06-26

**Authors:** Kevin E. Agner, Michael C. Larkins

**Affiliations:** 1 East Carolina University Brody School of Medicine, 600 Moye Blvd, Greenville, NC 27834 USA; 2 The Ohio State University College of Medicine, 370 W. 9 Avenue, Columbus, OH 43210 USA; 3 Department of Emergency Medicine, Wright State University, 2555 University Blvd, 110, Dayton, OH 45324 USA

**Keywords:** Adamantinoma, Surgical Oncology, Pediatric Oncology, Cancer Epidemiology

## Abstract

Adamantinoma (AD) is a rare bone cancer accounting for less than 0.1–0.5% of all primary bone tumors. No consensus guidelines exist for the treatment of this disease and long-term (twenty-year) survival has yet to be explored. The Surveillance, Epidemiology, and End Results (SEER) Program was queried for patients with a diagnosis of primary AD (ICD-O-3 code 9261/3). Demographic and treatment variables were analyzed via Fisher’s Exact Test and 20-year overall survival (20y OS) was assessed via log-rank analysis. Seventy-four patients with AD were identified; median age was 20–24 years. Multivariate analysis demonstrated that patients < 25 years of age at diagnosis with AD had increased 20y OS compared to those > 24 years (HR = 0.28; p = 0.028), while no other variables influenced survival. Subanalysis demonstrated patients > 40 years saw decreased survival (46% [11%, 81%]) compared to those ≤ 40 years (96% [89%, 104%]; p = 0.005). Patients ≤ 40 years of age at diagnosis were more likely to have local disease (78% of all 49 local cases) and less likely to have distant disease (0% of two cases) compared to patients > 40 years (p = 0.017). Stratifying by surgical procedure, no difference in 20y OS was appreciated (p = 0.12). Younger age at diagnosis provides mortality benefit and increased proportion of localized disease for those diagnosed with AD. No other demographic or treatment variables were found to influence 20y OS. Population-based analysis of AD is limited both by disease rarity and incomplete coding within SEER.

## Introduction

AD is an exceedingly rare bone cancer, accounting for less than 0.1–0.5% of all primary bone tumors [1]. The most common anatomic site is the long tubular bones of the leg, often located mid-shaft in the tibia or fibula [2]. Diagnosis is usually between the ages of 20–50 years, and patients typically present with a painful, swollen mass which can be palpated. Subsequent biopsy demonstrates nests of epithelial-like cells with a background of fibrous stroma [3]. Imaging techniques such as X-rays, CT scans, MRI scans, and bone scans are used for differential diagnosis, determining the size, and locating the tumor [2, 4]. Surgical excision is the standard treatment for adamantinomas, aiming for wide resection with limb reconstruction to minimize recurrence [5]. Chemotherapy and radiotherapy are generally considered ineffective in the management of this disease, though no consensus treatment guidelines exist given the low incidence [6]. Adequate interventional margins are a priority as local recurrence is a concern, and metastasis occurs at rates as high as 18–32% primarily to lymph nodes or lungs [1]. However, studies show optimistic five- and ten-year survival rates upwards of 90% [7].

Population-based studies are crucial for understanding the epidemiology, treatment patterns, and survival outcomes of AD due to its rarity [8]. Currently, there is merely one international multicenter study including adamantinomas [9], and one epidemiological survey utilizing the Surveillance, Epidemiology, and End Results (SEER) Program database for AD outcomes and survival [7]. Neither of these studies assessed twenty-year survival despite high reported survival rates and both studies analyze patients diagnosed at a minimum of five years before the writing of this article. The primary objective of this study was to assess the demographic factors, treatment modalities, and prognostic indicators that influence patient outcomes.

## Materials and Methods

### Patient Identification

The National Cancer Institute’s Surveillance, Epidemiology, and End Results (SEER) Program was used to identify patients with a diagnosis of AD. The 17-registry Incidence data set with cases diagnosed between 2000 and 2020 was selected [10]. Inclusion criteria were International Classification of Diseases (ICD)-O-3 histology/behavior code 9261/3 corresponding to AD “of the long bones;” codes 9351/1 (corresponding to adamantinomatous craniopharyngioma) and 9261/3 (corresponding to osteofibrous dysplasia-like AD) were not included. Surgical codes were determined based on the 2021 SEER Staging Manual [11].

### Data Analysis

Data was analyzed in SPSS (Version 29.0; Armonk, NY: IBM Corp.), with a p-value of < 0.05 as the cutoff for statistical significance and 95% confidence intervals reported in brackets [95% CI]. Multivariate analysis was conducted via Cox regression based on twenty-year overall survival (20y OS). Categorical variables were compared via two-sided Fisher’s Exact Test. Kaplan–Meier (KM) survival curves were generated and compared using log-rank analysis for univariate and subanalysis.

## Results

### Cohort Overview

**Fig. 1:**
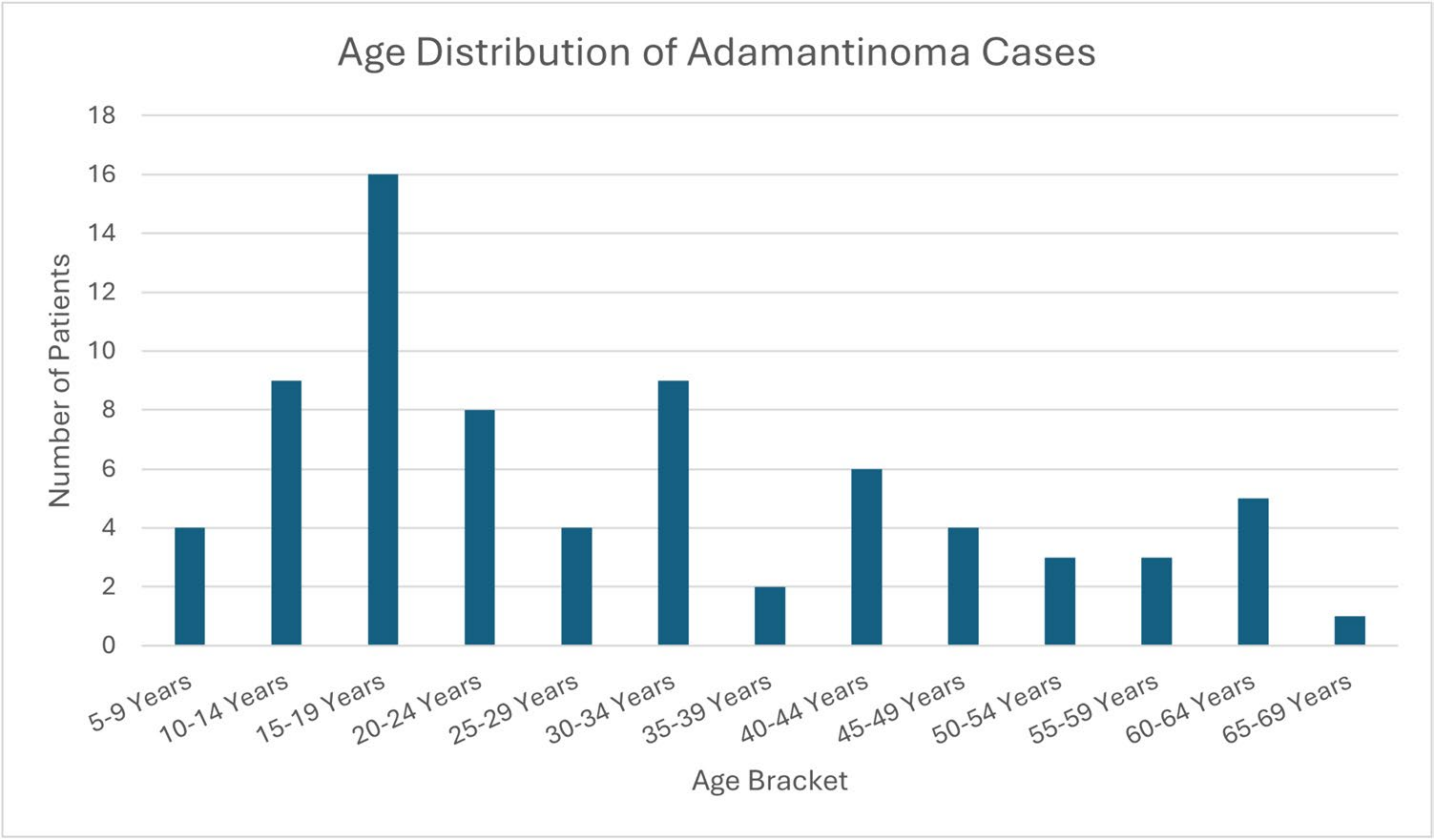
Age at Diagnosis of AD. Graph depicting the age at diagnosis of AD for patients identified between 2000 and 2020 identified via the SEER Program

Seventy-four patients with AD were identified. All patients had complete survival information and all cases had disease classified as malignant. Median age bracket was 20–24 years of age at diagnosis. Age, months from diagnosis to treatment, and household income were split into bivariate groups to allow for strong statistical comparison. A bar graph displaying the age distribution of diagnoses can be found in Fig. 1. One patient had disease diagnosed radiographically without microscopic confirmation, all others (n = 73) had histologically confirmed disease. 97.3% of all cases were diagnosed as AD of the lower limb; the remainder were AD of the long bones (including the scapula). Demographic and disease characteristics can be found in Table 1.

**Table 1:** Demographic and Disease Characteristics of Patients Diagnosed with Adamantinoma in the US between 2000 and 2020

Variable	Number (% of Cohort; n = 74)
*Age at Diagnosis (Two Groups)*	
10–24 Years	37 (50.0%)
25–69 Years	37 (50.0%)
Age at Diagnosis (Three Groups)*	
< 20 Years	29 (39.2%)
20–39 Years	23 (31.1%)
> 40 Years	22 (29.7%)
*Year of Diagnosis*	
Before or During 2008	36 (48.6%)
After 2008	38 (51.4%)
*Sex*	
Male	34 (45.9%)
Female	40 (54.1%)
*Race*	
Asian/Pacific Islander	8 (10.8%)
Black	7 (9.5%)
White	57 (77.0%)
Unknown	2 (2.7%)
*Disease Site*	
Long Bones of the Upper Limb	2 (2.7%)
Long Bones of the Lower Limb	72 (97.3%)
*Summary Stage*	
Local	49 (66.2%)
Regional	6 (8.1%)
Distant	2 (2.7%)
Unstaged/Incomplete Staging	17 (23.0%)
*Grade*	
I (Well-differentiated)	10 (13.5%)
II (Moderately Differentiated)	6 (8.1%)
III (Poorly Differentiated)	1 (1.4%)
IV (Undifferentiated)	0 (0.0%)
Unknown/Incomplete Grading	57 (77.0%)
*Median Household Income*	
< $75,000	33 (44.6%)
≥ $75,000	41 (55.4%)

^*^Age at Diagnosis was divided into three groups as a part of survival subanalysis

Demographic and disease characteristics for patients diagnosed with AD identified via the SEER Program. Median household income was reported as inflation adjusted as of 2021

Treatment information regarding the identified group of patients can be found in Table 2.

**Table 2:** Treatment Characteristics of Patients Diagnosed with Adamantinoma in the US between 2000 and 2020

Variable	Number (% of Cohort; n = 74)
*Laterality*	
Left	35 (47.3%)
Right	39 (52.7%)
*Time from Diagnosis to Treatment (Months)*	
< 2 Months	44 (59.5)
≥ 2 Months	23 (31.1%)
Unknown	7 (9.5%)
*Treatment*	
No Treatment	7 (9.5%%)
Surgical Treatment Only	66 (89.2%)
Surgery + Chemotherapy*	1 (1.4%)
*Surgical Procedure*	
None	7 (9.5%)
Local Tumor Destruction/Partial Resection	12 (16.2%)
Radical Surgery with Limb Salvage	51 (68.9%)
Major Amputation	4 (5.4%)

^*^Unknown if this was adjuvant or neoadjuvant chemotherapy

Treatment characteristics for patients diagnosed with AD identified via the SEER Program. Surgical codes were determined based on the SEER 2021 Staging Manual Appendix C: Surgery Codes [11]. Local surgery and partial resection procedures were based on codes 15–26, radical surgery with limb salvage corresponded to code 30, and codes 51–54 were classified as major amputation. Information on the specific chemotherapeutic regimen administered was not available

### Multivariate Analysis

Cox regression analysis demonstrated an increased 20y OS among patients diagnosed between 10 to 24 years of age (Hazard Ratio (HR) = 0.28, p = 0.028); all other variables did not demonstrate an impact on survival. Results can be found in Table 3. Variables with > 20% unknown or blank entries were excluded from this analysis to protect data integrity (specifically disease Grade and stage).

**Table 3:** Cox Regression Analysis of 20-year Overall Survival among Patients with Adamantinoma

Variable	Hazard Ratio	p-value
10–24 Years / 25–69 Years*	0.28	0.028
Sex	3.15	0.25
Race	N/A	0.57
Household Income (< $75,000 / ≥ $75,000)	0.45	0.43
Laterality	0.68	0.71
Time from Diagnosis to Treatment	N/A	0.36
Surgical Treatment Y / N	0.00	0.99

^*^Age at diagnosis with AD

Results from multivariate Cox regression analysis. Overall p-value is listed for categorical variables with more than two categories

### Prognostic/Disease Trends Based on Age

**Fig. 2:**
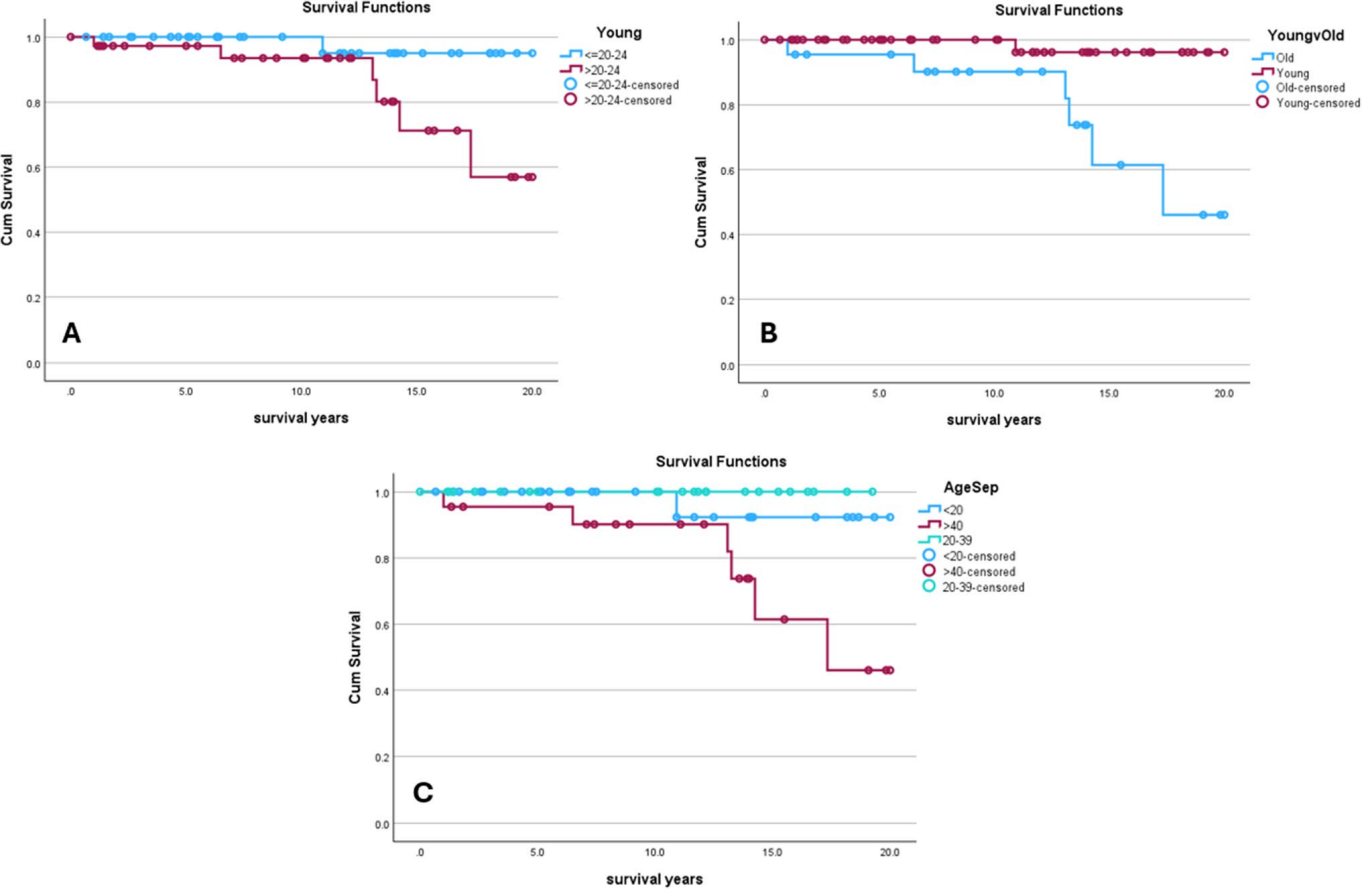
Age-stratified 20y Overall Survival (20y OS) Analysis of Patients with AD. Kaplan–Meier subanalysis of patients diagnosed with AD between 2000 and 2020 in the US based on age. **2A:** Comparison in 20y OS between patients ≤ 20–24 years of age at diagnosis (n = 37; 95% [85%, 100%]) versus those > 24 years (n = 37; 57% [25%, 89%]; p = 0.052). **2B:** Comparison between patients > 40 years of age at diagnosis, denoted “Old” in the Fig. (20y OS = 46% [11%, 81%]) versus those ≤ 40 years, denoted “Young” in the Fig. (20y OS = 96% [89%, 100%]) demonstrated patients in the younger group had increased survival (p = 0.005). **2C:** Comparison in 20y OS between patients divided into three groups: < 20 years at diagnosis (n = 29), between 20 and 39 years at diagnosis (n = 23), and ≥ 40 years at diagnosis (n = 22). Patients greater than 40 years at diagnosis had decreased 20y OS (46% [11%, 81%]) compared to both those less than 20 years at diagnosis (92% [78%, 100%]) and those diagnosed between 20 and 39 years of age (100% 20y OS; p = 0.017)

Based on the graph in Fig. 1, it can be seen that the majority of AD cases are diagnosed in younger patients, with a small but constant number diagnosed up to the 7th decade of life. Survival analysis based on age can be found in Fig. 2. Univariate analysis demonstrated no difference in survival between patients ≤ 24 years of age at diagnosis (20y OS = 95% [85%, 100%]) compared to those diagnosed at 25 years of age or greater (57% [25%, 89%]; p = 0.052). Further stratification into three distinct, approximately equal groups (age at diagnosis < 20 years, 20–39 years, and > 40 years) demonstrated patients greater than 40 years at diagnosis had decreased 20y OS (46% [11%, 81%]) compared to both those less than 20 years at diagnosis (92% [78%, 100%]) and those diagnosed between 20 and 39 years of age (100% 20y OS; p = 0.017; see Fig. 2C). Direct comparison between those > 40 years of age at diagnosis (20y OS = 46% [11%, 81%]) versus those ≤ 40 years (20y OS = 96% [89%, 100%]) demonstrated patients in the younger group had increased survival (p = 0.005). This was essentially a subgroup analysis performed between the oldest group of patients and the remainder in the cohort, and was done after the difference in 20y OS among the three age groups was seen to be driven by those aged > 40 years.

Using Fisher Exact tests, demographic analysis showed no difference among the cohort when separated into three age groups with respect to sex (p = 0.061), race (0.48), year of diagnosis (diagnosed before or during 2008 vs after 2008, giving an approximate equal bivariate split among all cases; p = 0.47), or household income (p = 0.38). No difference in the severity of disease was observed between these age groups (Grade: p = 0.13; stage: p = 0.33). No difference was found with regards to laterality (p = 1.00), months from diagnosis to treatment (p = 0.27), rates of surgery (p = 0.18), or types of surgery performed (p = 0.48).

However, performing the same analysis stratified by age at diagnosis > 40 years versus ≤ 40 years demonstrated patients ≤ 40 years were more likely to have local disease (78% of all 49 local cases) and less likely to have distant disease (0% of two cases) compared to patients > 40 years (p = 0.017). No difference seen between these groups with respect to sex (p = 0.38), race (p = 0.59), year of diagnosis (p = 0.18), Grade (p = 0.19), laterality (p = 0.52), frequency of surgical treatment (p = 0.074), surgical procedure (p = 0.21), time from diagnosis to treatment (p = 0.13), or median household income (p = 0.12).

### Demographic Subanalysis

On univariate analysis, no difference in 20y OS was seen with respect to sex (male: 74% [49%, 99%] versus female: 71% [37%, 100%]; p = 0.54). Similarly, comparison between White (n = 57) versus non-White (n = 15) showed no difference in 20y OS (White patients: 75% [52%, 99%] versus non-White: 73% [37%, 100%]; p = 0.85). Finally, no difference between patients with median household income of < $75,000 (84% [62%, 100%] compared to those with median household income ≥ $75,000 was seen (68% [39%, 97%]; p = 0.54).

### Grade and Stage Subanalysis

Limited analysis of 20y OS stratified by both Grade and stage was assessed. No difference in survival was found based on stratification by Grade among patients with AD (p = 0.38), though 77% of these patients had unknown Grading. No difference was seen among patients based on stage of disease (p = 0.36). Excluding patients with incomplete or unknown staging, no difference that met statistical significance was appreciated between cases with local, regional, and distant disease (n = 57; p = 0.20).

### Treatment Subanalysis

**Fig. 3:**
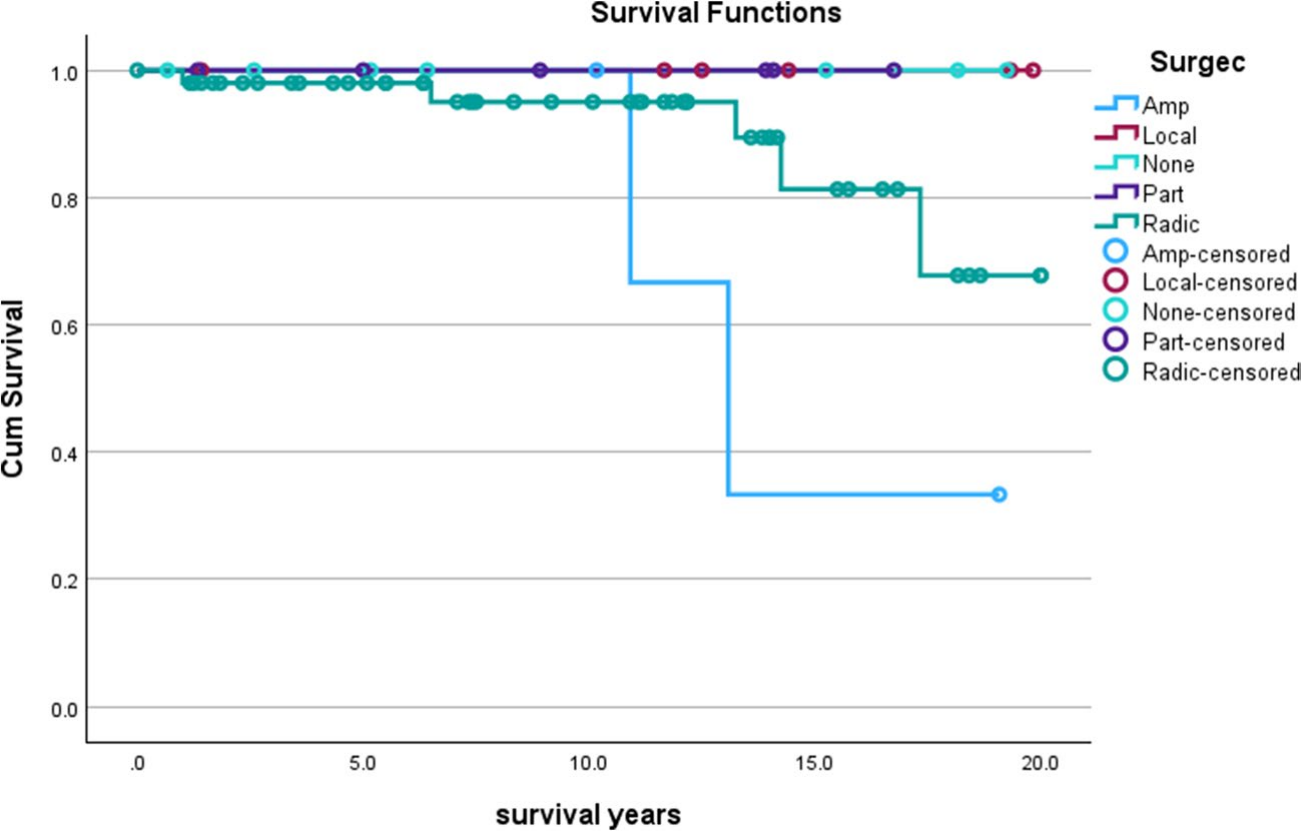
20y Overall Survival (20y OS) Analysis of Patients with AD Stratified by Surgical Procedure. Comparison of survival based on surgical procedure, with “Amp” referring to patients that underwent amputation, “Local” referring to patients that underwent local excision, “Part” referring to patients that underwent partial limb resection, “Radic” referring to patients that underwent radical resection with limb preservation, and “None” referring to patients that did not undergo surgery. No difference in 20y OS was seen among these patients (p = 0.12)

Comparison between treatment with or without surgery demonstrated no difference in 20y OS (p = 0.35). Stratifying by surgical procedure, no difference in 20y OS that met statistical significance was appreciated (p = 0.12; see Fig. 3). Further stratification by local disease stage demonstrated a similar trend, with no difference observed (p = 0.97), though analysis of other disease stage was limited secondary to low patient census.

No difference in survival was found with respect to laterality (right-sided disease 20y OS: 78% [57%, 99%] versus left-sided disease: 75% [45%, 100%]; p = 0.84). Time from diagnosis to treatment did not impact 20y OS: excluding patients with unknown time from diagnosis to treatment (n = 7), patients with time to treatment (TTT) < 2 months (20y OS = 68% [36%, 100%] had no difference in survival compared to those with TTT ≥ 2 months (76% [47%, 100%]; p = 0.64). No association was found between TTT and patient age (≥ 40 years of age at diagnosis (n = 22) versus those < 40 years (n = 52); p = 0.43) or between TTT and the surgical procedure utilized for treatment (p = 1.00). Furthermore, no difference was seen among the relative frequency of patients having metastatic disease and TTT (p = 0.76).

## Discussion

This analysis of AD in the US identified 74 patients diagnosed between 2000–2020. AD predominantly affects younger individuals, with a median age at diagnosis of 20–24 years. Multivariate Cox regression analysis revealed an increased 20y OS among patients 24 years of age or younger at diagnosis compared to those older than 24 years (HR 0.28, p = 0.028). Subanalysis revealed that patients over 40 years of age at diagnosis exhibited decreased 20y OS compared to younger age groups (46% and 96% OS, respectively; p = 0.017). Furthermore, younger patients (< 40 years at diagnosis) were more likely to have localized disease and less likely to have distant metastasis compared to older patients (those > 40 years at diagnosis; p = 0.017). This significant difference in disease localization among younger AD patients likely contributes to their higher survival rates and favorable prognostic outcomes. Younger age in general may contribute to the increased survival among those with a disease with an already favorable prognosis.

However, it is important to note the underreporting of disease grade, with 77% of cases grouped in “Unknown/Incomplete Grading.” Similarly, 23% of patients in this analysis had incomplete staging, which could be secondary to a presumption of local disease (perhaps in the context of younger patients without systemic symptoms and a lesion(s) identified on imaging). A similar thought process may be information the underreporting of disease Grade in SEER. No significant difference in 20y OS was observed between patients treated with or without surgery (p = 0.99), and stratification by surgical procedure did not reveal any differences in survival as well (p = 0.12). This may be secondary to the high survival rate already present among those with AD, and the difference in surgical procedure may have implications for patient morbidity not assessed by the SEER Program currently. Time to treatment (TTT) did not impact survival, which may be a reflection again of the high survival associated with AD or could be a reflection of the limited number of cases treated more than two months after diagnosis: only 12 of the 74 cases identified had a TTT greater than two months.

The largest published multicenter study on AD reported 139 cases of classic AD (as opposed to the osteofibrous dysplasia-like variant). Notably, a decreased incidence of local recurrence in females versus males (HR = 0.57, p = 0.018) was seen [9]. One key difference in this study compared to the one presented in this paper is that patient data were obtained from tertiary bone tumor centers, as opposed to a national, population-level cancer database. This allowed for the collection and analysis of detailed treatment information. Schutgens et al. report that all cases were histopathologically confirmed in this analysis, though also report that given the extended period over which samples were collected (between 1985 and 2015), the quality of such diagnoses were variable. Additionally, while Schutgens et al. performed analysis based on the treatment, margins, recurrence, and presence of metastasis among patients with classic AD, no analysis based on disease stage or Grade was carried out. It is unclear whether this information was not available or excluded.

A 49-patient long-term follow-up study conducted between 1939 and 2012 corroborates these sex-based differences [12]. This study found that male AD patients had an increased risk of local disease recurrence with a HR of 5.92 (p = 0.04), but found no significant association between sex and mortality or metastasis [12]. A SEER-based study also examined survival differences associated with sex [7]. Aytekin et al. reported a 100% 10-year survival rate for 43 females and an 84.8% rate for 49 males. However, it did not utilize a multivariate test for further statistical analysis. Comparatively, our study found no difference in AD incidence with respect to sex on multivariate analysis (p = 0.25); stratifying age into three groups (age at diagnosis < 20 years, 20–39 years, and > 40 years) did not demonstrate a difference in incidence among sexes either (p = 0.061).

There are limitations inherent to this study, including the underreporting of disease grade, its retrospective nature, and the relatively small sample size. Lack of consistent coding for disease Grade and stage further limited the level of analysis that could be conducted. Furthermore, a newer variant of Ewing sarcoma, adamantinoma-like Ewing sarcoma, has been reported in the literature [13–15]. Currently the SEER Program does not include a specific ICD-O-3 code for this variant, and it is possible some cases of AD listed include this variant of Ewing sarcoma. The sample size of 74 patients limited the power of the statistical analysis, especially when stratifying for various factors and expecting robust statistical significance. Additionally, the SEER database has limitations concerning the data provided, particularly regarding the specifics of disease treatment, patient morbidity, and metastasis. One patient was diagnosed via radiographic rather than histologic methods, creating the possibility of this patient being misdiagnosed, though the radiographic diagnosis of adamantinoma is established in the literature [5]. Future studies with larger cohorts, comprehensive pathological evaluation, and improved surgical documentation are warranted.

## Data Availability

The data and materials that supported the findings of this study are available from the corresponding author upon reasonable request. Original data are available at Surveillance Epidemiology and End Results (SEER) database (https://seer.cancer.gov/data/).
