# Correction to: Adamantinoma: A SEER-based Epidemiological Analysis

**DOI:** 10.1007/s13193-025-02442-1

**Published:** 2025-11-15

**Authors:** Kevin E. Agner, Michael C. Larkins

**Affiliations:** 1 East Carolina University Brody School of Medicine, 600 Moye Blvd, Greenville, NC 27834 USA; 2 The Ohio State University College of Medicine, 370 W. 9 Avenue, Columbus, OH 43210 USA; 3 Department of Emergency Medicine, Wright State University, 2555 University Blvd, 110, Dayton, OH 45324 USA

**Correction to: Indian Journal of Surgical Oncology (December 2024) 15(4):809–815**

**https://doi.org/10.1007/s13193-024-02002-z**

The original version of this article unfortunately contained a mistake.

The disclaimer statement was missing from this article and should have read 'The views and opinions expressed in this article/presentation are those of the author(s) and do not necessarily reflect official policy or position of the United States Air Force, Defense Health Agency, Department of Defense, or U.S. Government.'.

The original article has been corrected.

